# Primary progressive apraxia: an unusual ideomotor syndrome

**DOI:** 10.1186/s40734-017-0064-0

**Published:** 2017-11-14

**Authors:** Yeva M. Fernandez, Steven J. Frucht

**Affiliations:** Mount Sinai, 5 East 98 street 1st floor, New York, NY 10029 USA

**Keywords:** Apraxia, Tauopathy, Ideomotor

## Abstract

**Background:**

Primary progressive apraxia is a rare form of apraxia in the absence of dementia which develops insidiously and is slowly progressive. Most reports of patients with apraxia also describe coexisting aphasias or involve additional apraxias with affected speech, usually in the setting of neurodegenerative diseases such as corticobasal degeneration, Alzheimer’s disease or frontotemporal dementia. The aim of this report is to describe and demonstrate by video two cases of isolated primary progressive ideomotor apraxia seen in our clinic.

**Case presentation:**

We describe two patients with 2–5 years of progressive difficulty using their hands, despite having intact cognition and lack of correlating lesions on imaging.

**Conclusion:**

We report two cases of primary progressive apraxia that may be early presentations of taupathic disease in both patients. In both cases, there is isolated profound ideomotor apraxia of the hands, with preserved cognition, language skills, muscle power and tone, and gait. There are no correlating lesions on imaging.

**Electronic supplementary material:**

The online version of this article (10.1186/s40734-017-0064-0) contains supplementary material, which is available to authorized users.

## Background

Apraxia is a disruption of ability to perform skilled movements in the absence of sensory, motor or language deficits. Primary progressive apraxia is a rare form of apraxia which develops insidiously in the absence of dementia, and is slowly progressive. Personality, behavior and comprehension are preserved. Originally, the concept was described by Mesulam in 1982 in patients who demonstrated development and slow progression of aphasia in the absence of dementia [1, 2]. Primary progressive apraxia is usually a presenting symptom of a neurodegenerative disease, as reported by Fukui et al. [3].

There are many types of limb apraxias, including ideational/conceptual apraxia, and limb kinetic apraxia, but this case report will focus mainly on the ideomotor apraxia seen in our patients [4]. Ideomotor apraxia refers to a disconnection between the motor system and stored information essential to coordinating, sequencing and organizing appropriate movements relative to space, sometimes referred to as an engram [5]. The concepts of tool utilization and learned skills are preserved, however there is failure to carry out specific goal oriented movements, or the goal is met with irregular movements [5, 6]. In addition, there is a dissociation between the ability to carry out tasks voluntarily versus involuntarily [6]. Radiologic investigations in the few reported cases of primary progressive apraxias and in cases of primary progressive aphasias generally show left hemispheric atrophy and involvement of the dominant parietal lobe [5, 7, 8].

## Case presentations

### Case 1

A 72- year- old right handed retired hairdresser presented with 5 years of progressive isolated difficulty with hand movements. He first noticed his symptoms while trying to style his wife’s hair. He began having difficulty with tasks involving fine motor movement, such as belt buckling, tying shoe laces and buttoning shirts. His left hand dexterity was worse than the right. He had difficulty using eating utensils, and was unable to voluntarily smile or laugh. At the time of his initial visit, he was unable to write, could not draw out letters, but could read and comprehend fully. MRI of the brain showed moderate cerebral atrophy, chronic right frontal cortical infarcts and white matter ischemic changes. A DaT scan was normal. The patient was tried on both donepezil and memantine which did not yield improvement. Neuropsychiatric testing was done, but we did not have results for our own review.

On our examination (Additional file 1: Video S1), he was alert and oriented to time, place and person. He could name objects and his speech was fluent. He was also able to repeat and to follow complex commands that did not involve the use of his hands. Strength was full throughout, tone was normal, balance was normal and coordination testing with finger to nose was intact. He could identify his fingers by pointing to the correct finger with the contralateral hand, but he was unable to lift the identified finger. Despite being able to identify all his fingers, he was unable to show 2 fingers on the left hand, tap his fingers or to mimic hand postures. He had preserved knowledge of how to use tools such as a scissor and stapler (Additional file 2: Video S2). When asked to pantomime movements such as brushing his teeth and brushing his hair, he used his body parts as objects. He attempted to write, but could not form letters on the page or copy simple figures. His apraxia of the hands was asymmetric, with more difficulty on the left. He also had apraxia of vertical gaze and of saccadic initiation. Despite preserved ability to spontaneously smile or laugh, he was unable to smile or to laugh on command.



**Additional file 1: Video S1.** This video demonstrates the patient’s exam. The patient explains that he is barely able to write as he cannot form letters. When trying to copy the word “TODAY”, his letters are poorly formed, the size of each letter varies, and he requires considerable time to copy each letter. He also has difficulty copying an image of a cube and of a diamond. He can identity the 4th digit on his left hand, but has difficulty manipulating the finger. Although he tries several times, he cannot do finger taps on the left hand and cannot mimic hand movements. He is able to track for smooth pursuit testing but cannot look up on command, and cannot smile on command. (MPG 165630 kb)

**Additional file 2: Video S2.** This video demonstrates the patient’s exam. The patient demonstrates that he has intact ability to use tools including a scissor, stapler and spoon. (MPG 41806 kb)


He was started on carbidopa/levodopa 25/100 1 tablet three times a day without benefit, and an FDG PET was obtained showing decreased metabolism in the frontal lobes, basal ganglia and brainstem. It also showed central volume loss, suggestive of a neurodegenerative process.

### Case 2

A 72-year-old right handed woman developed difficulty using her hands at age 70. She first noticed problems moving her hands while typing at work. The difficulty was asymmetric, mainly involving her left hand. Her struggle with typing progressed to difficulty manipulating a toothbrush to brush her teeth, holding eating utensils, and bathing. Her condition progressed, resulting in inability to use both hands, requiring assistance with all activities. She described knowing what she wanted her hands to do, but was unable to make her hands comply. She had not experienced any changes in speech, cognition, or balance.

On our examination, (Additional file 3: Video S3) she was alert and oriented. Language, prosody, and comprehension were intact. Cranial nerves were intact and motor strength was full in all extremities. Coordination and balance were also intact. On motor exam, her voice was slightly hypophonic, but speech was normal. Eye movements were normal. There was mild rigidity in her left wrist. She had normal speed of movement in her legs, but her hand movements were severely impaired. She was unable to pantomime and had body utilization when asked to pantomime brushing her teeth. In addition, she was unable to show an “OK” sign or a salute. Testing for rapid alternating hand movements and opening and closing of hands was difficult as she could not coordinate holding her hands forward while opening and closing them. Sensory modalities including two-point discrimination, stereognosis, and graphesthesia were intact.


Additional file 3: Video S3. This video demonstrates the patient’s exam. She is unable to voluntarily move her right or left hand or show 2 fingers. She is unable to properly salute and has body utilization when asked to show how she would brush her teeth or brush her hair. She has profound difficulty controlling her hands with hand dexterity testing and when performing rapid alternating movements. (MPG 73970 kb)


Following her initial evaluation, she was started on carbidopa/levodopa 50/200 1 tablet three times per day. On her follow-up visit, she had discontinued the medication as it caused imbalance and allodynia. Her symptoms remained unchanged. An FDG- PET scan was normal.

## Discussion

This report describes two patients with isolated, insidious, progressive, debilitating inability to use their hands over a period of 2–5 years. The patients in these cases have an isolated profound ideomotor apraxia mainly involving movement of the hands, with preservation of speech and cognition. Few cases of primary progressive apraxia have been reported in the literature. Since the first documented case of primary progressive aphasia, published by Mesulam in 1982, there been multiple documented cases of primary progressive aphasia, but only a few documented cases of primary progressive apraxia [1].

Ideomotor apraxia is subtype of apraxia characterized by a failure to perform a goal oriented movement due to disruption in the organization of timing, spatial orientation and sequence of the movement. Patients demonstrate abnormal speed of movement, disarray of sequenced movements and abnormal amplitude of movement. They may also substitute body parts as tools or objects when pantomiming. The concept of tool use is intact, however the ability to execute the use of the tool is affected where speed, amplitude and trajectory are more effortful and it may take multiple attempts to execute the goal. Pantomiming the use of the tool generally yields erroneous results, but when the tool itself is used, there is an improvement in the performance of an action. This is likely due to visual and kinesthetic cues provided by the tool that helps to direct appropriate posture and positioning to carry out the task. Despite the erroneous movements, the goal of the movement is recognizable. Generally, it is a disorder that affects limb movement bilaterally [4, 9].

Limb-kinetic apraxia is another subtype of limb apraxia that is mainly confined to the hands and fingers. Unlike with ideomotor apraxia, in limb-kinetic apraxia all finger movements are disrupted and unrecognizable. There are extraneous non-functional movements that are clumsy and nonproductive. The objective of the movements is unrecognizable. This type of apraxia is generally affects the limbs unilaterally. Both limb kinetic apraxia and ideomotor apraxia are not mutually exclusive. The clinical picture may be confusing, as it is possible to have both ideomotor apraxia and limb kinetic apraxia affecting a limb, as suggested by Leiguarda [10]. and seen in the patient described by Fukui et al. [3].

The concept of conceptual/ideational apraxia has been debated and there have been multiple interpretations of its definition. Most authors agree that it is exemplified by a loss of ability to appropriately use tools for a selective action. The patient may not recognize or select the correct tool for a given action or goal. Furthermore, there is a loss of appropriate sequencing of action to properly utilize a tool.

The patients we describe in this report experienced difficulty with fine motor movement of the hands as their initial symptom. Their difficulties with fine motor movements slowly progressed to a gross failure of purposeful hand movements. Both patients demonstrate an ideomotor apraxia where they have a clear understanding of how to produce an action, but the resulting action is incorrect or they are unable to manipulate their hands to produce the appropriate movements. They may also demonstrate errors of movements, such as body utilization [6].

Many tests have been developed for formal evaluation of apraxia. Dovern et al. reviewed various tests that have been designed as diagnostic tools for apraxia and experimental assessment for apraxia [11]. However, an in-depth discussion of these test sets is beyond the scope of this case report. Apraxias are generally characterized and organized into categories based on the types of error made by patients and by the suspected pathways involved. Leiguarda et al. reviewed the evaluation of limb praxis, involving testing both the praxis production system and the conceptual system [4]. In the case of our patients, we mainly tested the production system by evaluating the ability to carry out transitive (tool utilization) and intransitive (communicative gestures and non-representational tasks) movements. Our patients were unable to pantomime utilization of tools such as scissors or a toothbrush. When asked to demonstrate how to brush their hair they would use their hands as a comb. Furthermore, they were also unable to mimic simple hand gestures such as an “OK” sign or produce hand movements testing dexterity and coordination.

In keeping with the definition of ideomotor apraxia set by Zadikoff et al., our patients demonstrated bilateral hand involvement, and on imaging had diverse anatomical involvement, including the basal ganglia [6]. Patients categorized as having limb kinetic apraxia usually demonstrate a degenerative process in either the frontal or parietal lobes on imaging and have pathologically confirmed testing of a degenerative process in these anatomical areas.

A review of literature reporting patients with ideomotor apraxia suggests that much like our patients, the majority of affected patient have symptoms that initially involve hand movements. In their review, Kawamura et al. reviewed 35 cases of patients with primary progressive apraxia. Of the 35 patients, 22 of these cases had isolated involvement of the hands as a presenting symptom 7 of the 35 cases reported had involvement of upper and lower limbs as a presenting symptom. In rare cases, patients have been reported to initially present with gait abnormality [8], speech difficulty [3], and orofacial apraxia [12]. Interestingly, many of the patients with ideomotor apraxia, including our patients, had an asymmetry in the handedness of their symptoms, with the left hand being reported as the more severely affected hand [13]. It may be expected that the handedness of the symptoms would correlate with atrophy or lesions seen on imaging, however we did not observe this. In patient 1, there was no asymmetric right hemispheric atrophy or asymmetric hemispheric hypometabolism on PET scan to correlate with the severity of the apraxia. The patient in case 2 did not have any reported abnormalities in her PET scan to correlate with the asymmetric presentation of her hand clumsiness.

### Imaging

There are variable imaging findings in reported cases of apraxia and aphasia. The original six primary progressive aphasia patients seen by Mesulam did not have brain lesions on imaging. The apraxic patients we report also follow in a similar pattern of functional decline, with no focal lesions to explain the clinical symptoms.

In a case of slow progressive limb kinetic apraxia, Otsuki et al [14], described a patient with an MRI showing left parietal atrophy and left pre- and post-central gyrus atrophy. In the cases presented by Fukui et al. [3], the patients had focal atrophy of the left parietal lobe and in some cases, diffuse cortical atrophy. Another case, reported by Martinez de Souza et al. [8], described a woman with both ideational and ideomotor apraxia that presented with apraxic gait and progressive inability to dress or to use her hands. This patient’s brain MRI demonstrated atrophy of the left superior parietal lobe and supplemental motor area. The patient presented by Piccirilli et al. had bilateral parietal atrophy [7]. While the brain MRIs of these patients suggest that primary progressive apraxia can be localized to a degenerative process in the parietal lobe, our patients’ MRIs did not show focal areas of atrophy. Their imaging findings are in contrast to the models suggesting that ideomotor apraxia is due to injury of the left parietal lobe, where motor engrams are thought to be encoded [15].

Functional imaging, including positron emission tomography (FDG-PET) and single-photon emission computed tomography (SPECT) was also used to assess reported cases of primary progressive apraxia. As with brain MRI findings, the patients in previously published reports had PET and SPECT scans showing hypometabolism involving the left parietal lobe [3, 5, 13, 14]. In contrast, our patient in case 1 has multiple areas of hypometabolism, particularly in the anterior frontal and temporal lobes. There is also hypometabolism seen in the basal ganglia and brainstem, which may account for clinical parkinsonism manifested as hypomimia and impaired eye movements. Furthermore, this suggests that his primary progressive apraxia is the presenting symptom of an underlying neurodegenerative disorder. In the second patient presenting with hypophonia and mild rigidity, there were no FDG-PET findings.

### Associations with Tauopathy

Apraxia has been associated with various tauopathy-related neurodegenerative disorders, such as Alzheimer’s disease, Pick’s disease, progressive supranuclear palsy and corticobasal degeneration [3]. In developing diagnostic criteria for Parkinsonian disorders, Litvan et al. included the presence of ideomotor apraxia a predictor for a diagnosis of corticobasal degeneration. However, their conclusions were based a on study population which may have underestimated the presence of dementia [16]. In the 10 cases of corticobasal degeneration reported by Riley et al., they observed that ideomotor and ideational apraxia could be present without cognitive decline [2]. In those cases, the patients were mainly affected by the deficits caused by their apraxia. Our patients also follow a similar pattern of absent to minimal cognitive decline with progressive and profound loss of ability to utilize their hands to execute purposeful learned movements. While the suspicion for corticobasal degeneration is low in the patients we present, there is a suspicion for progressive supranuclear palsy in case 1 due to impairment of vertical eye movements. Our patient’s inability to move his hands or face voluntarily may serve to further demonstrate that a progressive apraxia may be an early presenting symptom of a tauopathy-related disease and can help to contribute to their diagnosis as an early sign of disease.

Pharr et al. argue that apraxia is more commonly seen in patients with corticobasal degeneration as opposed to progressive supranuclear palsy [17]. They found that patients with corticobasal degeneration have a greater severity of apraxia, leading them to conclude that testing for apraxia is critical to distinguishing between a diagnosis of progressive supranuclear palsy and corticobasal degeneration. While our patients do not have CSF or pathologically confirmed tauopathy, the patients in our cases do show an insidious and gradually worsening ideomotor apraxia in the absence of cognitive deficits. We suspect that their presentation may be an early presentation of a tauopathy-related disease.

Progressive apraxia is generally associated with tauopathic disease, but the mechanism of causality is unclear despite the reported cases of pathologically confirmed tauopathy. Fukui et al. has reported a patient with a similar presentation of a primary progressive apraxia of speech and hand movement as the first symptoms of pathologically confirmed Pick’s disease. Despite pathologic confirmation of diagnosis, the mechanism underlying apraxia has not been elucidated. Models have been developed to explain the pathways interrupted in these diseases, leading to apraxia. Leiguarda et al. have suggested that the integration of cortico-striatial inputs to the parietal and frontal lobes, where motor engrams are thought to be stored may be disrupted in patients with Parkinson’s disease or progressive supranuclear palsy [10]. They concluded that damage of the basal ganglia was not sufficient to cause apraxia, as patients tested in their “on” and “off” states did not show a difference in the scores for exams testing for apraxia severity. They suspect that a combination of basal ganglia and cortical damage may be a more reasonable explanation. Moreover, they favor the possibly of an underlying cortical pathologic process as an etiology for progressive apraxia.

## Conclusions

We report two unusual presentations of what are most likely neurodegenerative disorders, possibly tauopathies. In both cases, there is preserved cognition, language skills, muscle power and tone, and gait in the setting of profound progressive ideomotor apraxia. Imaging is remarkable in our two cases for a lack of any discernable pattern, specifically a lack of parietal lobe involvement. Both cases demonstrated a lack of response to carbidopa/levodopa.

## Abbreviations

CSF: Cerebrospinal fluid
FDG-PET: Fluorodeoxyglucose-positron emission tomography
MRI: Magnetic Resonance Imaging
PET: Positron emission tomography
SPECT: Single-photon emission computed tomography
